# Supplementary material for: 3D Chromatin Alteration by Disrupting β-Catenin/CBP Interaction Is Enriched with Insulin Signaling in Pancreatic Cancer
Source: Cancers (Basel). 2024 Jun 12;16(12):2202. doi: 10.3390/cancers16122202 (PMC11201718; doi:10.3390/cancers16122202)
Supplement: Supplementary file 1 [file cancers-16-02202-s001.zip › Zhou_PancInh_Suppl_V3.8.pdf]

3D Chromatin Alteration by Disrupting β-Catenin/CBP Interaction Is Enriched with Insulin Signaling in Pancreatic Cancer

Yufan Zhou, Zhijing He, Tian Li, Lavanya Choppavarapu, Xiaohui Hu, Ruifeng Cao,
Gustavo W. Leone, Michael Kahn, Victor X. Jin

Supplementary Material

Supplementary Figure S1. Cell growth curve with the treatment of ICG-001.

(**A**) Growth curve of PATC50 cells in various concentrations of ICG-001. (**B**) Growth curve of HPNE cells in various concentrations of ICG-001.

A

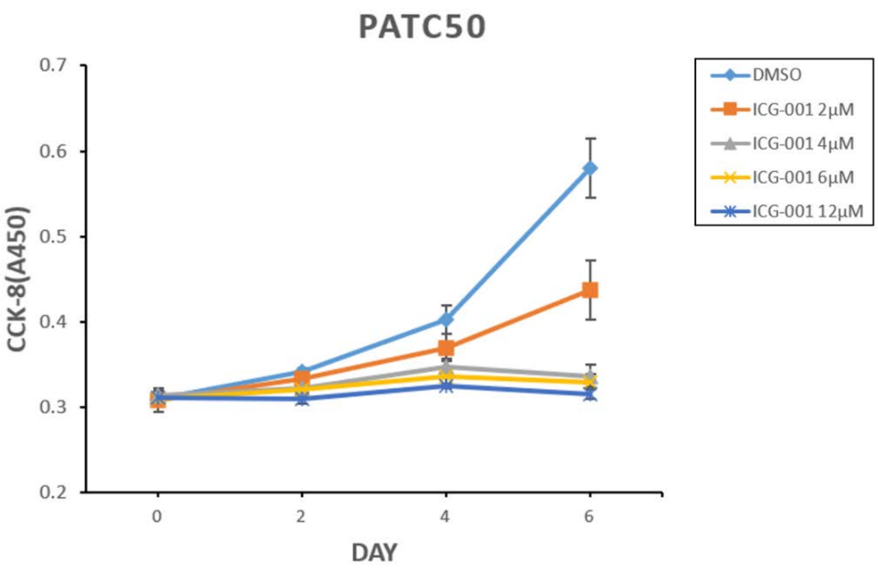

B

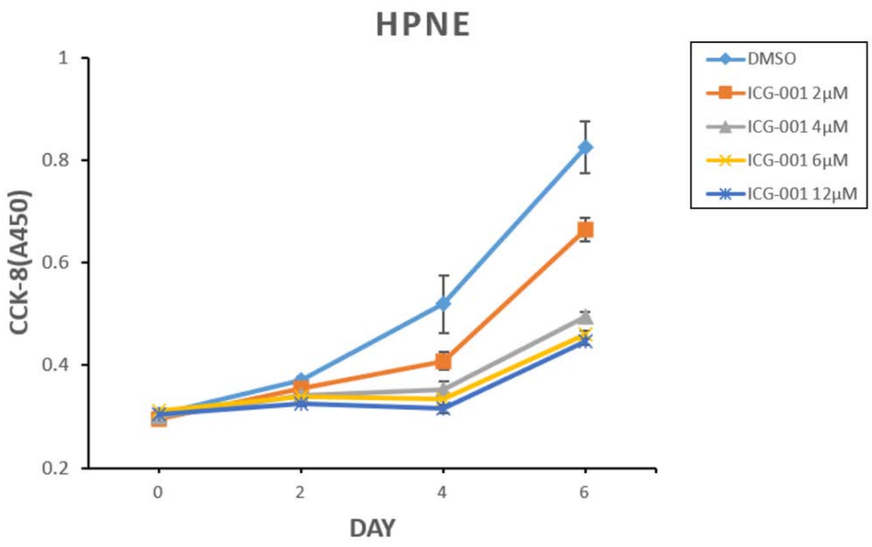

Supplementary Figure S2. Cell migration and invasion assays upon ICG-001 treatment.

(**A**) Cell migration and invasion assay for PATC50 in the presence of 10 μM ICG-001. (**B**) Cell migration and invasion assay for HPNE in the presence of 10 μM ICG-001.

A

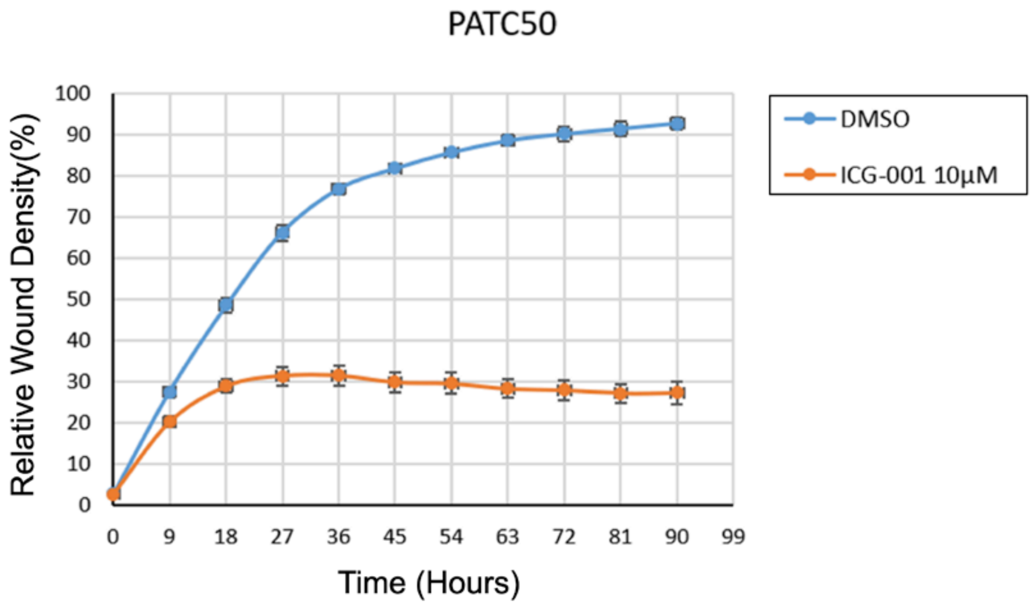

B

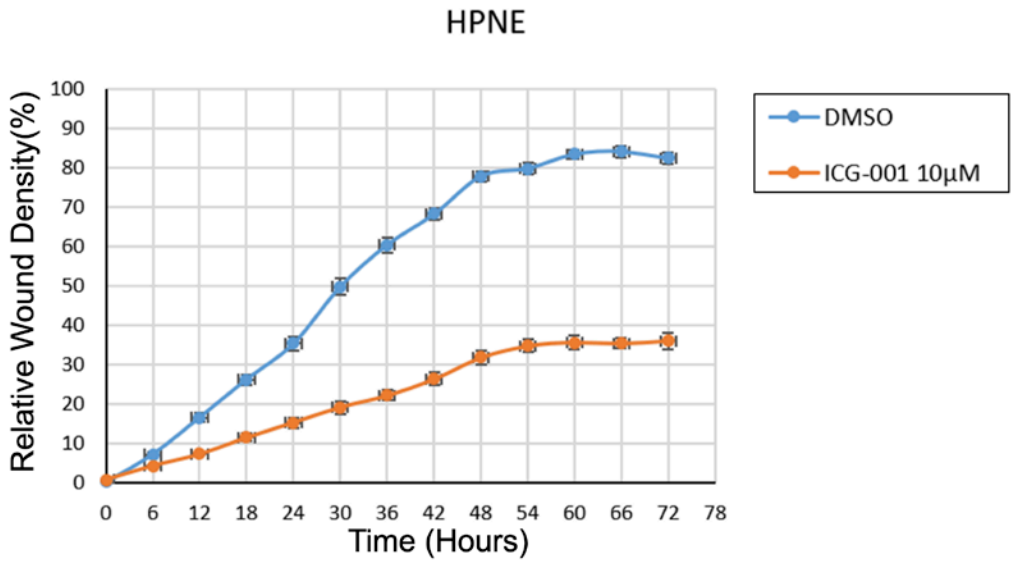

Supplementary Figure S3. Apoptosis analysis of cells with the treatment of ICG-001.

(**A**) Apoptosis analysis of PATC50 cells with ICG-001 treatment. (**B**) Apoptosis analysis of HPNE cells with ICG-001 treatment. * $p < 0.05$, student t test.

A

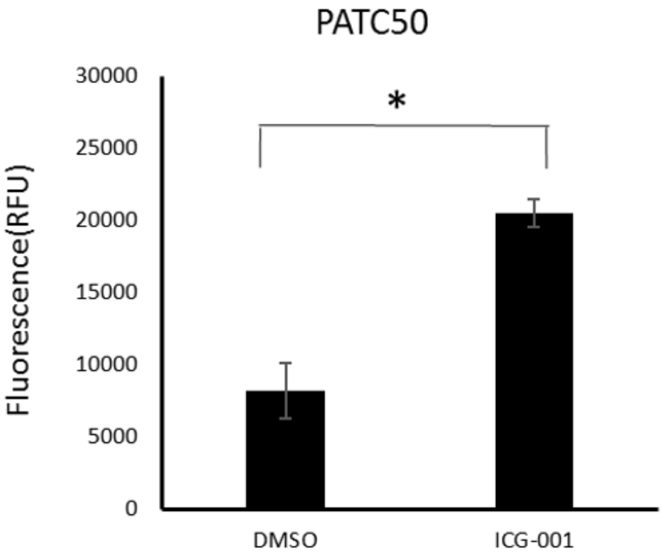

B

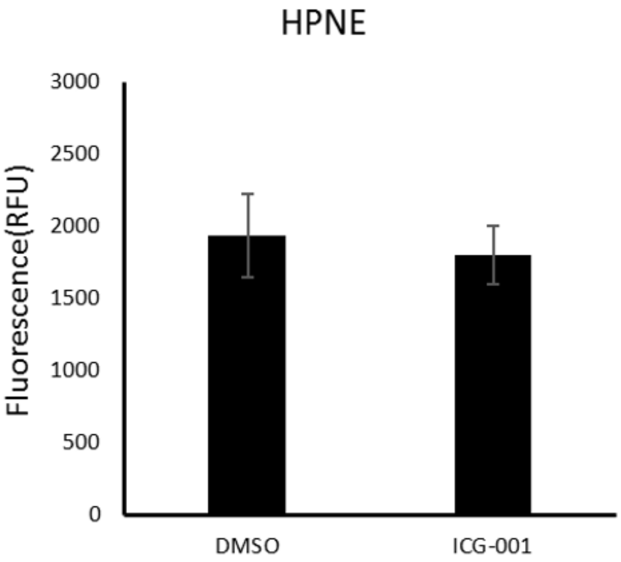

Supplementary Figure S4. Cell growth status with the combined treatment.

(**A**) Combination treatments of ICG-001 and Gemcitabine in PANC1 and PATC53. (**B**) Growth status of PATC50 cells with the combined treatment of ICG-001 and Gemcitabine. (**C**) Growth status of HPNE cells with the combined treatment of ICG-001 and Gemcitabine. I: ICG-001, G: Gemcitabine.

A

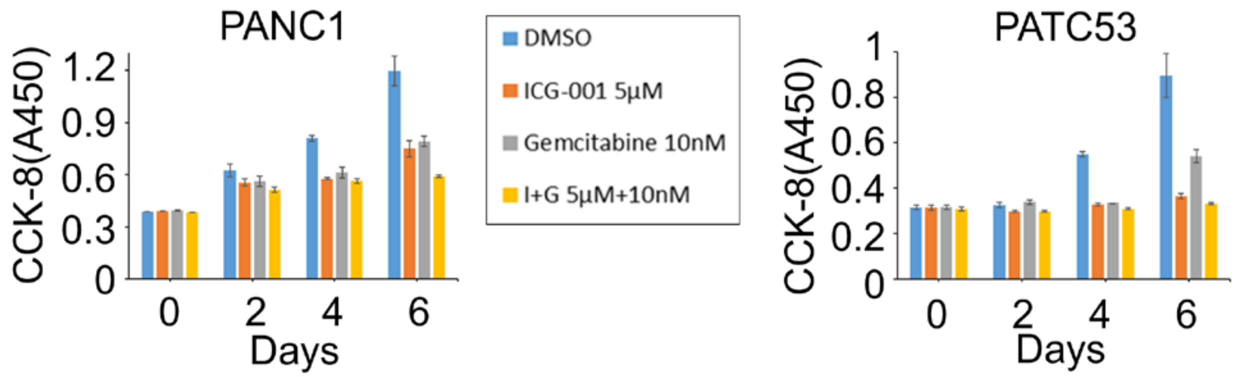

B

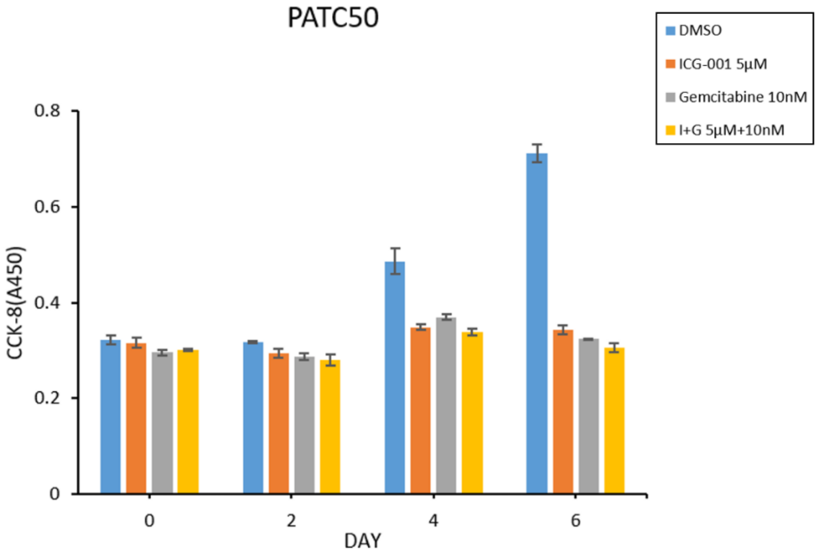

C

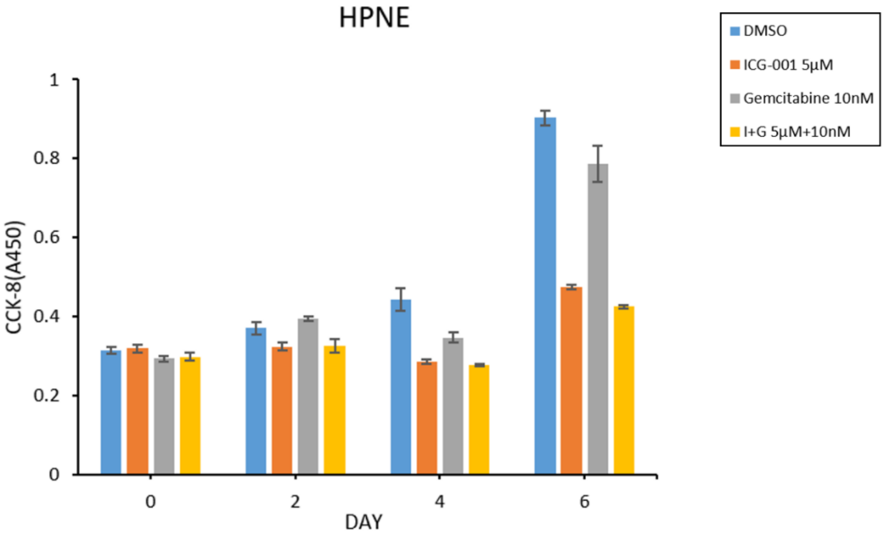
HPNE
DMSO
ICG-001 5μM
Gemcitabine 10nM
I+G 5μM+10nM
CCK-8(A450)
1
0.8
0.6
0.4
0.2
0
0
2
4
6
DAY

Supplementary Figure S5. The repeated Western blotting in PATC53 cells with the deletion of the enhancer region of IRS1.

(**A**) Three technical replicates of Western blotting were performed to show the expression levels of the downstream proteins in insulin signaling pathway. (**B**) No significant difference was showed by paired student t-test when Del-01 and Del-02 were treated with ICG-001 comparing to sgEmpty control. (**C**) Three technical replicates of Western blotting imaging gels for IRS1. (**D**). Three technical replicates of Western blotting imaging gels for the downstream proteins in insulin signaling pathway.

A

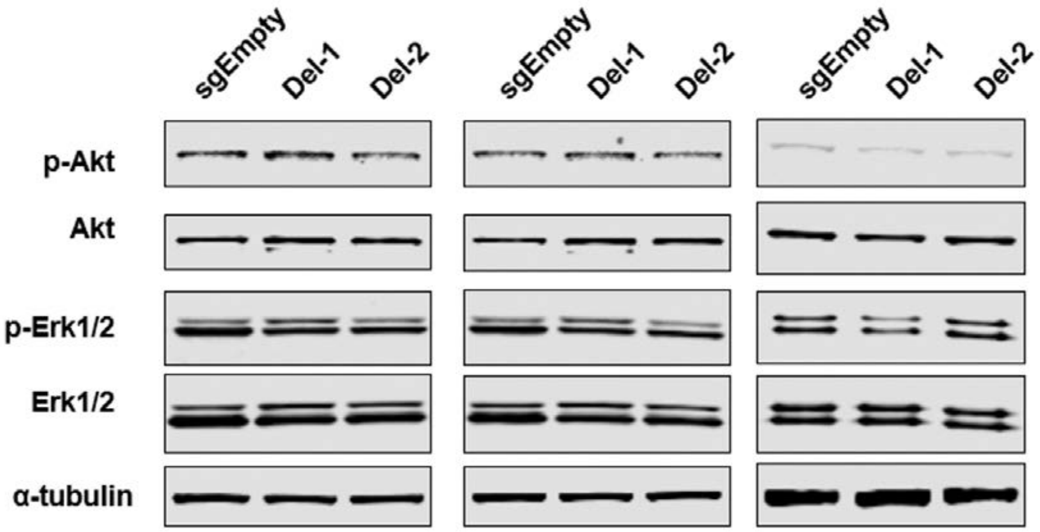

B

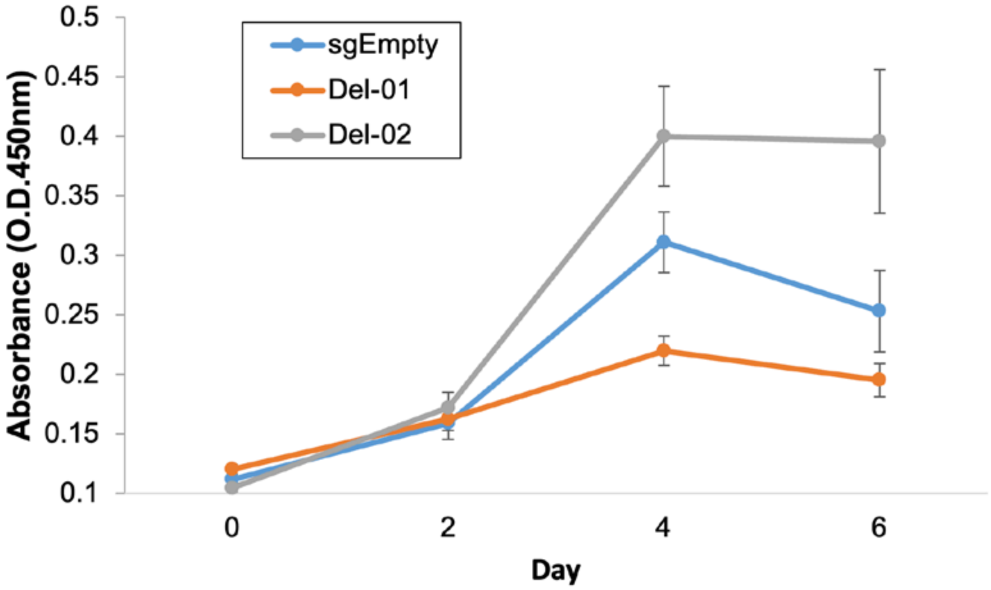

C

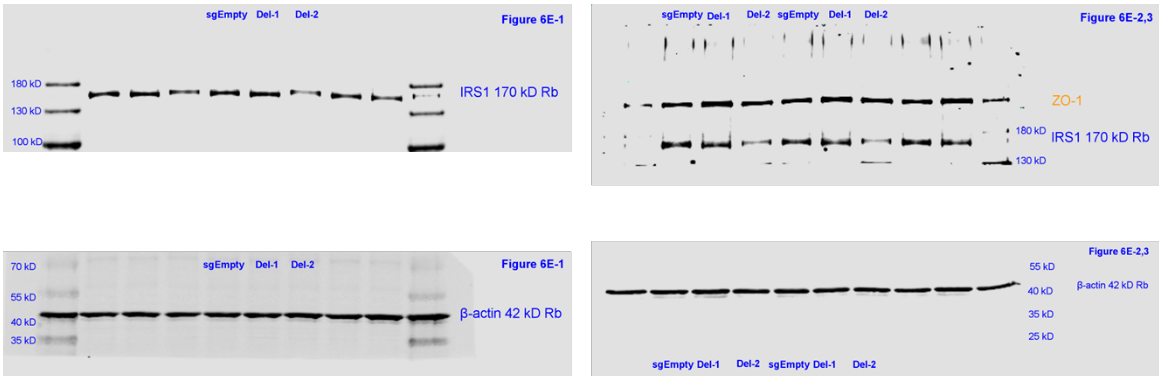
sgEmpty Del-1 Del-2
Figure 6E-1
180 kD
130 kD
100 kD
IRS1 170 kD Rb
sgEmpty Del-1 Del-2 sgEmpty Del-1 Del-2
Figure 6E-2,3
ZO-1
180 kD
IRS1 170 kD Rb
130 kD
70 kD
55 kD
40 kD
35 kD
β-actin 42 kD Rb
Figure 6E-2,3
55 kD
40 kD
35 kD
25 kD
β-actin 42 kD Rb
sgEmpty Del-1 Del-2 sgEmpty Del-1 Del-2

D

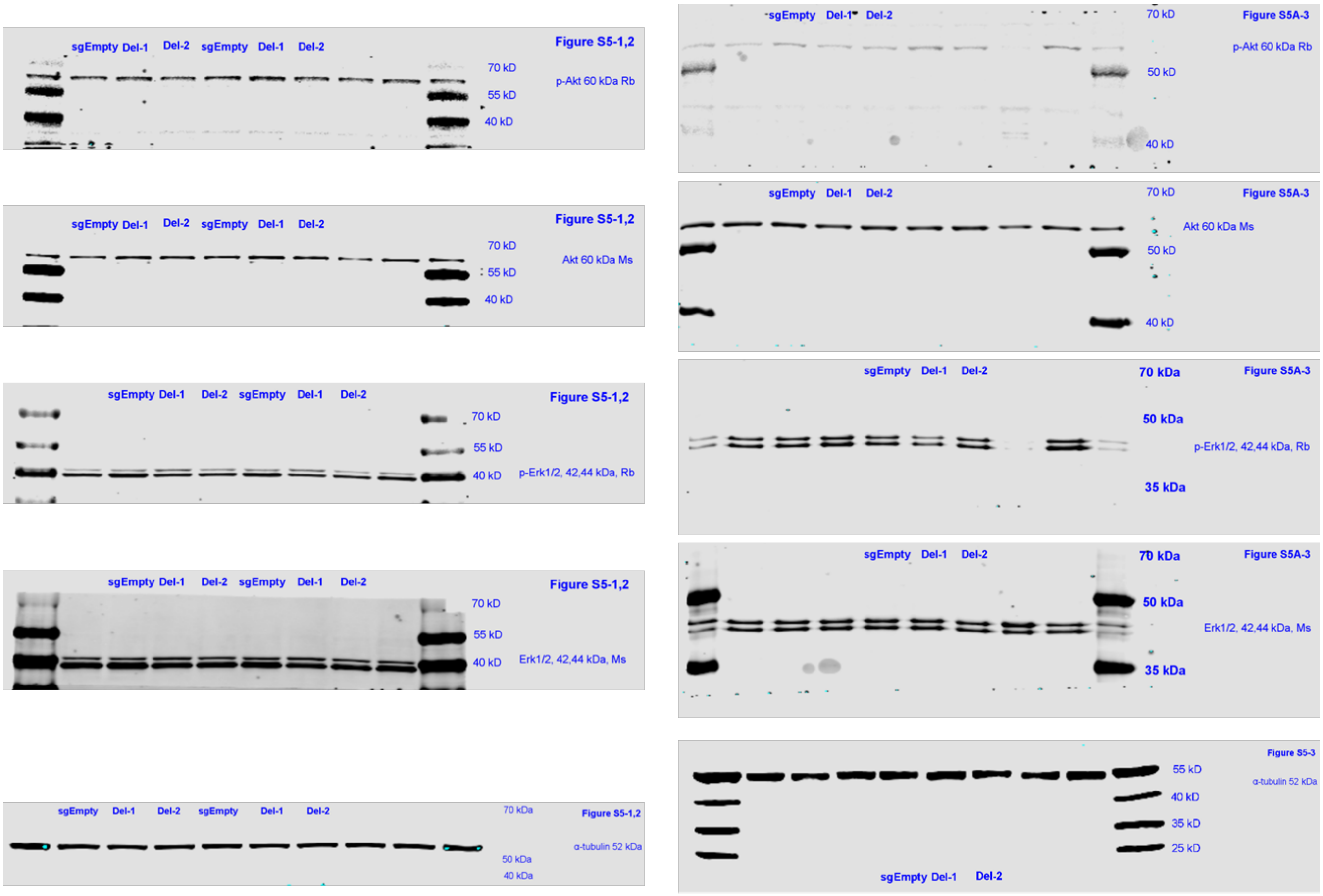
sgEmpty Del-1 Del-2 sgEmpty Del-1 Del-2
Figure S5-1,2
70 kD
55 kD
40 kD
p-Akt 60 kDa Rb
Akt 60 kDa Ms
p-Erk1/2, 42,44 kDa, Rb
Erk1/2, 42,44 kDa, Ms
70 kDa
50 kDa
40 kDa
α-tubulin 52 kDa
Figure S5A-3
50 kD
40 kD
50 kDa
35 kDa
Figure S5-3
55 kD
35 kD
25 kD

Supplementary Figure S6. RHBDD1 does not influence the cell growth and the mRNA level of IRS1 in PATC53 cells.

(**A**) RT-qPCR showed the mRNA expression level of RHBDD1 of the enhancer deletion clones (Del-01 and Del-02) decreased significantly. The deleted enhancer region of IRS1 was located in the coding region of the gene RHBDD1. (**B**) To check if the decreased level of RHBDD1 imfluences the the cell growth in PATC53 cells, shRNAs (shControl or shRHBDD1) were used to deplete the mRNA level of RHBDD1. (**C**) No significant differece of the cell growth curves was observed between PATC53 cells without or with RHBDD1 depletion using shRNA (shControl or shRHBDD1). (**D**) The mRNA level of IRS1 was not inhibited or increased in PATC53 cells with RHBDD1 depletion.

A

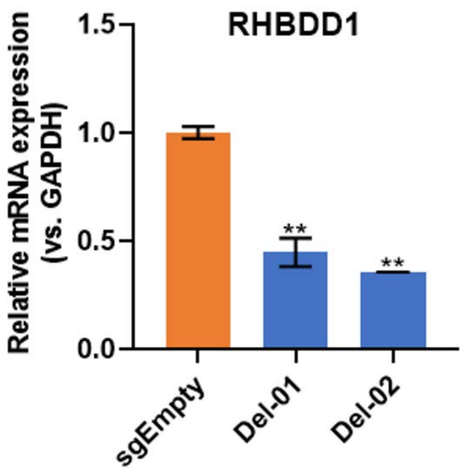

B

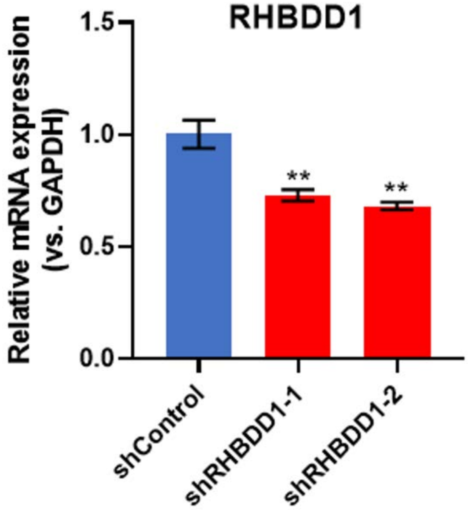

C

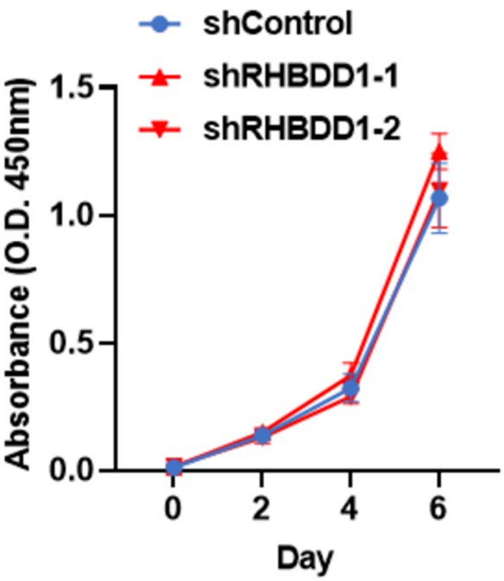
shControl
shRHBDD1-1
shRHBDD1-2
Absorbance (O.D. 450nm)
1.5
1.0
0.5
0.0
0
2
4
6
Day

D

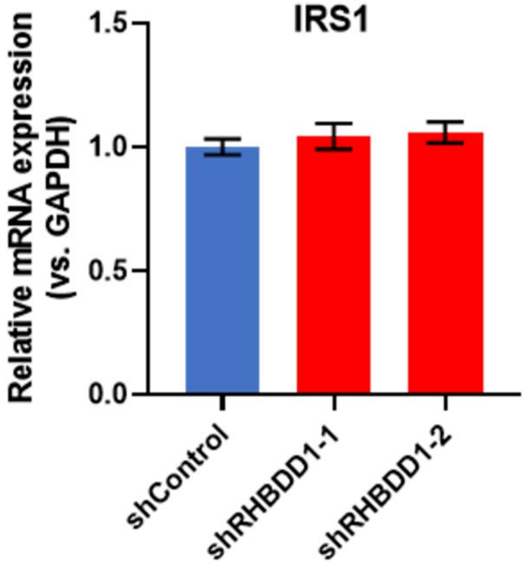
IRS1
Relative mRNA expression (vs. GAPDH)
1.5
1.0
0.5
0.0
shControl
shRHBDD1-1
shRHBDD1-2

Supplementary Table S1. Reads of Hi-C data

PANC1

	ENCODE PANC1	ENCODE PANC1	PANC1+ICG-001	PANC1+ICG-001
	Rep1	Rep2	Rep1	Rep2
Raw reads	151,103,112	137,874,940	138,855,403	160,655,787
Uniquely mapped	90,313,153 (59.80%)	67,559,617 (49.00%)	84,100,007 (60.60%)	96,896,977 (60.30%)
Valid Pairs	82,484,838 (91.30%)	62,661,214 (92.70%)	73,572,169 (87.50%)	87,263,424 (90.10%)
Removing duplicates	81,397,598 (98.68%)	56,817,809 (90.67%)	67,848,364 (92.22%)	79,953,474 (91.62%)

PATC53

	PATC53	PATC53	PATC53+ICG-001	PATC53+ICG-001
	Rep1	Rep2	Rep1	Rep2
Raw reads	335,546,131	334,793,995	338,795,426	284,332,904
Uniquely mapped	189,132,486 (56.40%)	178,395,348 (53.30%)	192,697,078 (56.90%)	136,147,287 (47.90%)
Valid Pairs	111,736,560 (59.10%)	69,126,040 (38.70%)	123,780,415 (64.20%)	74,636,388 (54.80%)
Remove duplicates	101,494,245 (90.83%)	62,896,779 (90.99%)	112,875,760 (91.19%)	71,342,891 (95.59%)

Supplementary Table S2. Reads of RNA-seq data

PANC1

	PANC1 Rep1	PANC1 Rep2	PANC1 Rep3	PANC1_ICG-001 Rep1	PANC1_ICG-001 Rep2	PANC1_ICG-001 Rep3
Raw Reads	33,744,666	46,629,164	37,984,529	38,716,394	45,844,799	38,864,612
Uniquely Aligned	28,340,835 (83.99%)	37,937,998 (81.36%)	30,678,396 (80.77%)	33,270,022 (85.93%)	36,975,473 (80.65%)	32,398,583 (83.36%)

PATC53

	PATC53 Rep1	PATC53 Rep2	PATC53 Rep3	PATC53_ICG-001 Rep1	PATC53_ICG-001 Rep2	PATC53_ICG-001 Rep3
Raw Reads	47,482,728	47,497,802	35,666,123	48,120,131	47,915,491	44,310,149
Uniquely Aligned	39,734,767 (83.68%)	38,895,089 (81.89%)	29,056,894 (81.47%)	40,314,629 (83.78%)	38,605,972 (80.57%)	35,973,406 (81.19%)

HPNE

	HPNE Rep1	HPNE Rep2	HPNE Rep3	HPNE_ICG-001 Rep1	HPNE_ICG-001 Rep2	HPNE_ICG-001 Rep3
Raw Reads	34,342,760	37,379,750	38,072,940	39,377,087	43,773,283	41,386,362
Uniquely Aligned	29,508,224 (85.92%)	31,116,071 (83.24%)	29,528,802 (77.56%)	32,458,878 (82.43%)	36,792,886 (84.05%)	33,944,167 (82.02%)

PATC50

	PATC50 Rep1	PATC50 Rep2	PATC50 Rep3	PATC50_ICG-001 Rep1	PATC50_ICG-001 Rep2	PATC50_ICG-001 Rep3
Raw Reads	46,551,843	37,445,665	40,955,126	46,845,888	44,896,703	35,538,038
Uniquely Aligned	39,037,119 (83.86%)	29,917,096 (79.89%)	31,082,866 (75.89%)	39,712,849 (84.77%)	36,694,714 (81.73%)	28,958,654 (81.49%)

Supplementary Table S3. List of sgRNA sequences and RT-qPCR primers

Oligonucleotides	Sequence	Experiment
IRS1 disDel-sgRNA-F-oligo1	caccgTGACCATGGAAGTTCTACCC	CRISPR-KO sgRNA design
IRS1 disDel-sgRNA-F-oligo2	aaacGGGTAGAACTTCCATGGTCAc	CRISPR-KO sgRNA design
IRS1 disDel-sgRNA-R-oligo1	caccgAGTAATTGCACTATACTACC	CRISPR-KO sgRNA design
IRS1 disDel-sgRNA-R-oligo2	aaacGGTAGTATAGTGCAATTACTc	CRISPR-KO sgRNA design
IRS1 disDel Validation-in-F	TTGCTGCTTTTCCTGTAAACCTTAG	CRISPR-KO validation primer
IRS1 disDel Validation-in-R	AAGCTCAACTGACAATGAGAAACTG	CRISPR-KO validation primer
IRS1 disDel Validation-out-F	TGGAATGCTGTTCCATTTAACTCAG	CRISPR-KO validation primer and Sanger seq
IRS1 disDel Validation-out-R	CCTTGGGGAGCTAGTGAAAACATA	CRISPR-KO validation primer
IRS1-Forward	ACTGGACATCACAGCAGAATGA	RT-qPCR
IRS1-Reverse	TCGTACCATCTACTGATGAGGAAG	RT-qPCR
RHBDD1-Forward	GTGCTGTAGGTTTCTCAGGAGTT	RT-qPCR
RHBDD1-Reverse	ACAGGAAAGCCCAAAATGTTGAC	RT-qPCR

Supplementary Table S4. Plasmids used in this study

Plasmid Name	Company	Catalog	Experiments
lentiCRISPR v2 plasmid	Addgene	#52961	CRISPR/Cas9 mediated distal region deletion
RHBDD1 shRNA Plasmid (h)	Santa Cruz Biotechnology	sc-94654-SH	shRNA
Control shRNA Plasmid-A	Santa Cruz Biotechnology	sc-108060	shRNA

Supplementary Table S5. List of 3C-qPCR primers

No	Name	Primer
1	EIF4EBP1 _Anchor	TATTTGAGTCTGTGGAGTCTTAAAAATATC
2	EIF4EBP1	GTATTAGGTTTCATTATACACATTTGAGAG
3	FBP1_Anchor	GACTCTGTCTGAAAAATAAAGAAATATAGC
4	FBP1	ATATATTTATGCTAGTTAGAGTTACGGTTC
5	FBP2_Anchor	CAAGTACAAGTCATGATAACACAATACATA
6	FBP2	ACATAAGGGAGAGTATAAGGGAGAGTATTA
7	IRS1_Anchor	CTTTTATCATACATGACAGTACCTTATACT
8	IRS1	AACTTCTAATATACAAGCATCTACTTACAC
9	KRAS_Anchor	TAGTAGATTAGTACACCACGTAGTATCTTT
10	KRAS	GTGTTTACAGTATTATTTAAGAAGCCTATG
11	MAPK9_Anchor	CAACTGATCTGTAACTAAGAATTCTACACA
12	MAPK9	CTGTTTATACATAAAGATACTTCTGAATGG
13	MKNK1_Anchor	CGTTCAGTAGACTTAAGATGGTTTAATAAT
14	MKNK1	GTATAACCACCACTATAATCAGAAATACAG
15	NRAS_Anchor	TTACTTTCTCTCCTCTTATTCCTTTAATAC
16	NRAS	CTTACTGAGAACTTAATTTTGAATGTCTAC
17	PIK3R1_Anchor	GTAAGTAGAGAGTGATGGTTATAACATTTG
18	PIK3R1	CTTGTATCTTGTAGACAAACTTGTAAAAAC
19	PPP1CB_Anchor	CTTAGAACTAAAAACCAAAGACTTCTACTT
20	PPP1CB	TCACAGTACTATTTTATTCAAGTGTCTAAC
21	PPP1R3A_Anchor	CTATTCATAGGCATAAAGAGATAATAAGGT
22	PPP1R3A	CTAAGTAAGTTATACGACATATTCATTTCC
23	PRKAA2_Anchor	CTGATGTTTAACTATGTGATCTATTTTGAG
24	PRKAA2	CCTACTACTTGAGTAAGTTATTTCACATCT
25	PRKAR1A_Anchor	GAATTATAGTAGATATTCTGGCTTTCTCTT
26	PRKAR1A	GCTAAAAGAGTCTCTCTCTAAGTATGTAAT
27	PRKAR1B_Anchor	ACTAGAAGTCAGTAACAGAAAGATAGATAG
28	PRKAR1B	CGATACTTGTAGGTGTAGTACAATTTTTAT
29	PRKCZ_Anchor	TATAGCAATGTGTAAAGAAGTGTGATTAAC
30	PRKCZ	AATACTGATGTTGGAAACTAGAAAACTATG
31	PTPN1_Anchor	AAAGTTTATAGGAGCTTTGTGAGTATAGTT
32	PTPN1	TACGTTTAGGTATGTTTAGAGACAGATACT
33	PYGM_Anchor	CTTCAGTTCTATATTACTTCTTTCTTTTCC
34	PYGM	CTTACTTTGATTGTATCATTAGAGTTGATG
35	RAF1_Anchor	AATTCAACTAGTTCACTATCTACAACAAAG
36	RAF1	CACTACACACTTATTCTAATAGCTAAAATC
37	GAPDH_Anchor	ATGCAAGGCTTTCTCTTAAATTAGC
38	GAPDH	AATTCTGAGCATTCTGTAGCAAACT

Supplementary Table S6. Antibodies used for Western blotting experiments

Antibody Name	Company	Catalog	Dilution
Rabbit monoclonal anti-IRS1	Abcam	ab40777	1:2000
Rabbit monoclonal anti-β-actin	Abcam	ab227387	1:5000
Mouse monoclonal anti-α-tubulin	Cell Signaling Technology	3873	1:2000
Mouse monoclonal anti-Akt (pan)	Cell Signaling Technology	2920	1:2000
Rabbit polyclonal anti-Phospho-Akt (Ser473)	Cell Signaling Technology	9271	1:2000
Mouse monoclonal anti-p44/42 MAPK (Erk1/2)	Cell Signaling Technology	4696	1:2000
Rabbit monoclonal anti-phospho-p44/42 MAPK (Erk1/2) (Thr202/Tyr204)	Cell Signaling Technology	4370	1:2000
